# Molecular Structure of the mRNA Export Factor Gle1 from *Debaryomyces hansenii*

**DOI:** 10.3390/ijms26041661

**Published:** 2025-02-15

**Authors:** Min Jeong Jang, Soo Jin Lee, Jeong Ho Chang

**Affiliations:** 1 Department of Biology Education, Kyungpook National University, 80 Daehak-ro, Buk-gu, Daegu 41566, Republic of Korea; 2 Department of Biomedical Convergence Science and Technology, Kyungpook National University, 80 Daehak-ro, Buk-gu, Daegu 41566, Republic of Korea; 3 Science Education Research Institute, Kyungpook National University, 80 Daehak-ro, Buk-gu, Daegu 41566, Republic of Korea

**Keywords:** mRNA export, nuclear pore complex, Dbp5, Gle1, IP6, *Debaryomyces hansenii*

## Abstract

Gle1 functions as a regulator of Dbp5, a DEAD-box-containing RNA helicase that is a component of the nuclear pore complex. In association with Gle1 and inositol hexakisphosphate (IP6), ADP-bound Dbp5 facilitates the release of RNA. The RNA-bound Dbp5 undergoes ATP hydrolysis and is activated by Gle1 in the presence of IP6. The formation of a ternary complex involving Dbp5, Gle1, and the nucleoporin Nup159 promotes ADP secretion and prevents RNA recombination. To date, several complex structures of Gle1 with its binding partners have been described; however, the structure of unbound Gle1 remains elusive. To investigate the structural features associated with complex formation, the crystal structure of N-terminally truncated Gle1 from *Debaryomyces hansenii* (*Dh*Gle1ΔN) was determined at a resolution of 1.5 Å. The *Dh*Gle1ΔN protein comprises 13 α-helices. Structural comparisons with homologs, all of which have been characterized in various complexes, revealed no significant conformational changes. However, several distinct secondary structural elements were identified in α1, α3, α4, and α8. This study may provide valuable insights into the architecture of yeast Gle1 proteins and their interactions with Dbp5, which is crucial for understanding the regulation of mRNA export.

## 1. Introduction

In eukaryotic cells, transcribed mRNA must be exported from the nucleus through nuclear pores for subsequent translation in the cytoplasm [1,2,3]. This export process requires the precise selection of mRNA by the nuclear pore complex (NPC) to ensure the exclusion of aberrant mRNAs [4,5]. Within the nucleus, properly processed mRNA interacts with various proteins in the NPC during export. During this process, certain mRNA-bound proteins are prevented from being transported by the RNA helicase DEAD-box protein 5 (Dbp5), which is located in the cytoplasmic region of the NPC [4,6]. A pivotal aspect of mRNA export is the dissociation of proteins from the messenger ribonucleoprotein complexes (mRNPs), which is believed to play a role in directing the export [2,4]. Consequently, only mRNAs are released into the cytoplasm. However, the regulation of mRNA remodeling remains not completely understood [2,4,6].

The superfamily 2 (SF2) RNA helicase Dbp5 is a DEAD-box protein found in a wide range of prokaryotic and eukaryotic organisms [2,4,6,7]. Dbp5 binds to RNAs in an ATP-dependent manner [8]. ATP facilitates the interaction between two distinct domains of Dbp5: the N-terminal domain (NTD) and the C-terminal domain (CTD), which in turn regulates RNA binding activity. Moreover, within the NPC filaments, Dbp5 is essential for the dissociation of the mRNA export receptor Mex67 and the RNA-binding protein Nab2 from the mRNP complex, a process that is dependent on its ATPase activity [2,8,9]. The Dbp5–Gle1–Nup159 complex orchestrates mRNA export in the NPC, utilizing various factors, including ATP, ADP, and the mRNP [2,4,6,10]. The RNA-bound Dbp5 acts through ATP hydrolysis, a process facilitated by both Gle1 and the small molecule inositol hexakisphosphate (IP6) [2,4,11,12]. A recent report indicated that the ATPase activity of Dbp5 is not activated by either tRNA or double-stranded RNA alone; rather, it necessitates the concurrent presence of Gle1 for activation [13].

Gle1 interacts with both the NTD and the CTD of Dbp5. The presence of ADP-bound Dbp5, in conjunction with Gle1 and IP6, facilitates RNA release by promoting the separation of these two domains. When this separation occurs, the Dbp5-NTD engages with the N-terminal region of Nup159, further enhancing the disconnection of the two domains [14]. The formation of the ternary complex consisting of Dbp5, Gle1, and Nup159 not only promotes the secretion of ADP but also prevents RNA recombination and encourages enzyme recycling [6,15]. While Nup159 is not associated, it facilitates the dissociation of ADP from Dbp5 within the NPC by inducing a conformational change in Dbp5 [15]. Although the structure of Gle1 and its ternary complex with Dbp5 and Nup159 has been elucidated in *Saccharomyces cerevisiae*, the precise mechanism underlying the mRNA export cycle remains not completely understood [4,6].

To investigate the Dbp5-mediated mRNA export cycle, we focus on the structure and function of Gle1. Several structures of Gle1 have been reported to date, including its binary complex with Dbp5 and its ternary complexes with both Dbp5 and Nup142, as well as Dbp5 and Nup159 [3,6,16]. However, the structure of Gle1 alone has yet to be characterized. To better understand the structural features associated with complex formation, we determined the crystal structure of Gle1 from *Debaryomyces hansenii* at a resolution of 1.5 Å. This structure was extensively compared to those of its structural homologs, including eIF4G. The findings of this study may provide insights into the molecular structure of Gle1 in fungal species, thereby expanding our understanding of its function.

## 2. Results

### 2.1. Overall Structure of DhGle1ΔN

**Figure 1:**
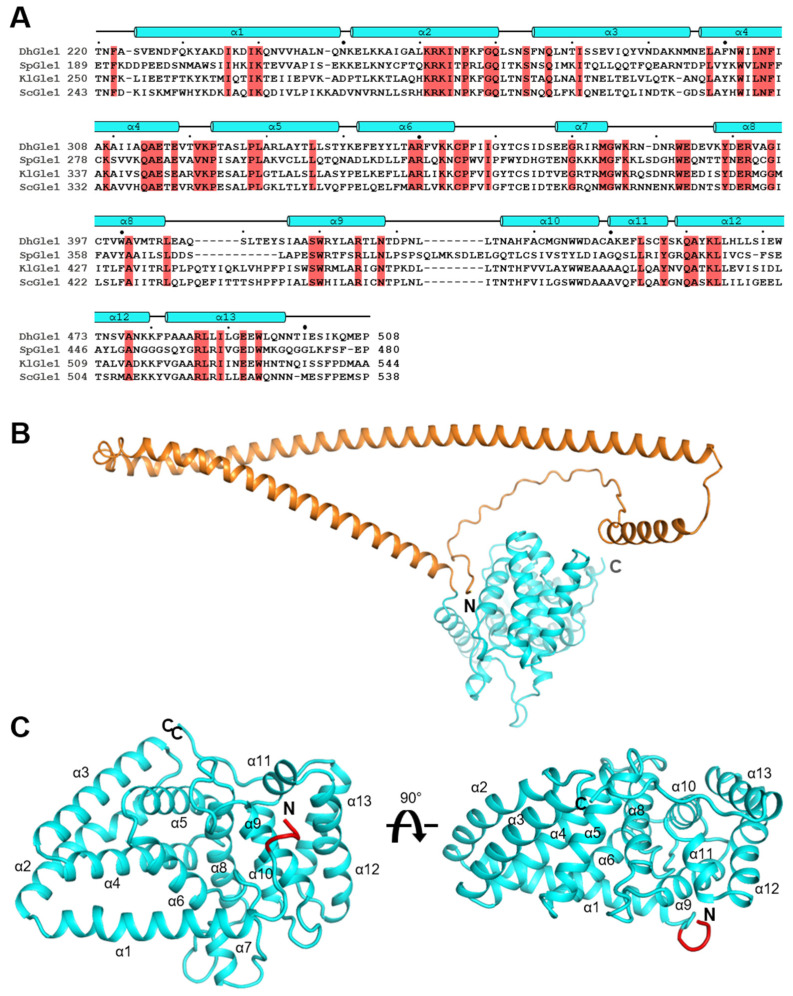
Overall structure of N-terminally truncated Gle1 from *Debaryomyces hansenii*. (**A**) An amino acid sequence alignment of four N-terminally truncated Gle1 proteins is shown: *Dh*Gle1 from *Debaryomyces hansenii*, *Sp*Gle1 from *Schizosaccharomyces pombe*, *Kl*Gle1 from *Kluyveromyces lactis*, and *Sc*Gle1 from *Saccharomyces cerevisiae*. Conserved residues with 100% identity among all four proteins are highlighted in red. Small and large black dots above the sequences are placed every ten and fifty residues, respectively. (**B**) The predicted structure of full-length Gle1 by Alpha-Fold 2 is presented by ribbon diagram. The N-terminal truncated region (residue 1–219) is colored in orange. (**C**) The overall structure of the N-terminally truncated Gle1 from *D. hansenii* (*Dh*Gle1ΔN), characterized in this study, is presented as a ribbon diagram. The N-terminal extra segment is colored red.

Expression of full-length Gle1 in *Escherichia coli* leads to inclusion body formation. To obtain a protein suitable for crystallization, we constructed a truncated form of *Debaryomyces hansenii* Gle1 (residues 220–508, *Dh*Gle1ΔN) in which the N-terminal region was removed from the protein (Figure 1A). The excluded N-terminal region is comprising three α-helices containing two long helices based on a predicted full-length Gle1 structure that possibly interacts with several nucleoporins other than Dbp5, and does not fully interact with Dbp5 [3,17,18,19] (Figure 1B).

The structure of *Dh*Gle1ΔN belongs to the space group *P*2_1_2_1_2_1_, and there is one molecule in the asymmetric unit. The monomeric *Dh*Gle1ΔN structure consists of 13 α-helices (Figure 1C), revealing that Gle1 comprises an all-α-helical HEAT repeat protein that interacts with both RecA-like domains of Dbp5 [6].

**Figure 2:**
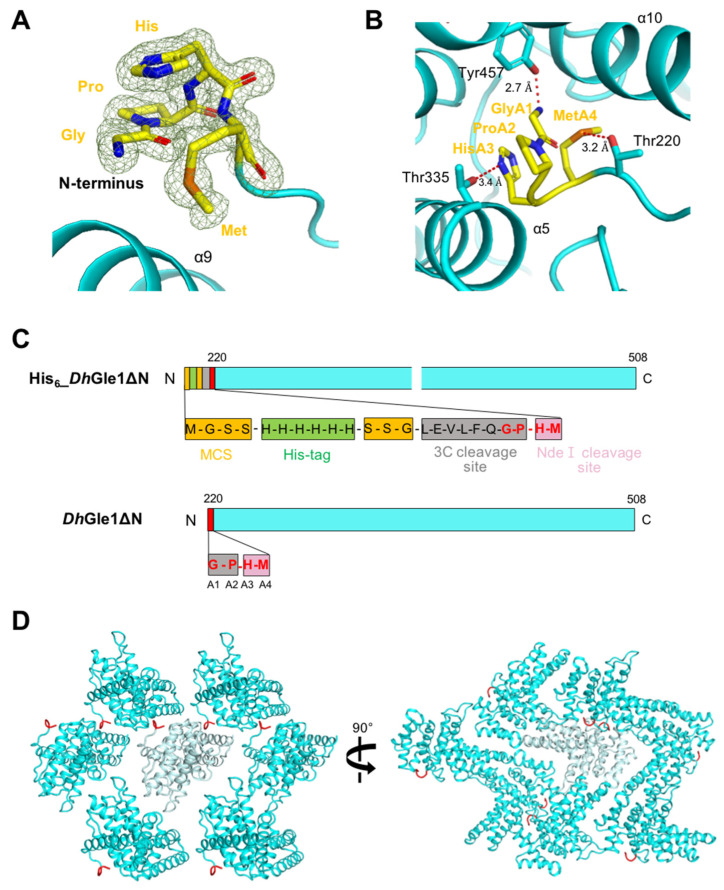
N-terminal extra segment and crystallographic packing of *Dh*Gle1ΔN. (**A**) Electron density representation of the N-terminal extra segment, which consists of Gly-Pro-His-Met residues. (**B**) A detailed view of the N-terminal extra segment (in yellow), showing the hydrogen bonds (illustrated as red dotted lines) it forms with surrounding residues. The bonding distances are provided for each bond. (**C**) A simplified depiction that includes detailed information regarding the long and short N-terminal extra segments of His6_*Dh*Gle1ΔN and *Dh*Gle1ΔN. (**D**) Crystallographic packing of *Dh*Gle1ΔN, presented from different perspectives rotated by 90° along the *y*-axis, with the N-terminal extra segment colored in red.

Interestingly, an extra N-terminal segment consisting of four residues—Gly-Pro-His-Met—was clearly shown in the electron density (Figure 2A). These residues, labeled A1–A4, were stabilized by Thr220, Thr335, and Tyr457 through hydrogen bonds (Figure 2B). Specifically, GlyA1, HisA3, and MetA4 interact with Tyr457, Thr335, and Thr220, respectively. To investigate the origin of this extra N-terminal segment, we analyzed the modified pET28a vector containing a 3C protease cleavage site. This analysis revealed that the multiple cloning site (MCS) of the vector includes a hexahistidine tag, the 3C cleavage site (Leu-Glu-Val-Leu-Phe-Gln-/Gly-Pro), and an Nde I restriction site (His-Met) (Figure 2C, upper panel). Upon cleavage of the histidine tag by the 3C protease, the four residues Gly-Pro-His-Met remained (Figure 2C, lower panel). Thus, while His6_*Dh*Gle1ΔN contained a 23-amino acid artificial N-terminal segment, *Dh*Gle1ΔN featured only the four additional N-terminal residues present in the untagged recombinant protein.

To explore the presence of the N-terminal extra segment in the structure, we analyzed the crystallography packing status. Each segment of the monomer is stabilized by neighboring molecules, fitting well into the space between them (Figure 2D, Supplementary Figure S1). In contrast, the His6_*Dh*Gle1ΔN molecules did not pack tightly together in the crystal structure due to their shape and the presence of the longer N-terminal segment including the hexahistidine tag, resulting in a poor X-ray. The crystallographic packing pattern suggests that the extended N-terminal segment may inhibit stable interactions between adjacent molecules. Taken together, these findings indicate that the N-terminal tag plays a critical role in determining the quality of the crystals.

### 2.2. Structural Comparison of DhGle1ΔN with Its Homologs

Homologous structures of *Dh*Gle1ΔN were identified using the DaLi web-based server, which facilitated systematic structural comparisons with molecules in the Protein Data Bank [20]. The top three identified homologs were Gle1 in complex with Dbp5 from *S. cerevisiae* (*Sc*Gle1ΔN; PDB ID: 3PEV), Gle1 in complex with Nup42 GBM from *Homo sapiens* (*Hs*Gle1ΔN; PDB ID: 6B4F), and Gle1 in complex with Nup42 GBM from *Chaetomium thermophilum* (*Ct*Gle1ΔN; PDB ID: 6B4H), with root mean square deviations (r.m.s.d.) of 1.2, 2.3, and 2.2 Å, and Z scores of 41.1, 29.8, and 29.5, respectively (Table 1). The three homologous Gle1 structures were compared based on their equivalent regions in relation to *Dh*Gle1ΔN. Notably, *Dh*Gle1ΔN displayed structural similarity to the eukaryotic translation initiation factor eIF4G, which serves as a platform for the SF2 DEAD-box ATPase eIF4A from *S. cerevisiae* (Sc_eIF4GΔN in complex with *Sc*_eIF4A, PDB ID: 2VSX), showing a root mean square deviation (r.m.s.d.) of 3.9 Å and a Z score of 15 (Table 1).

**Figure 3:**
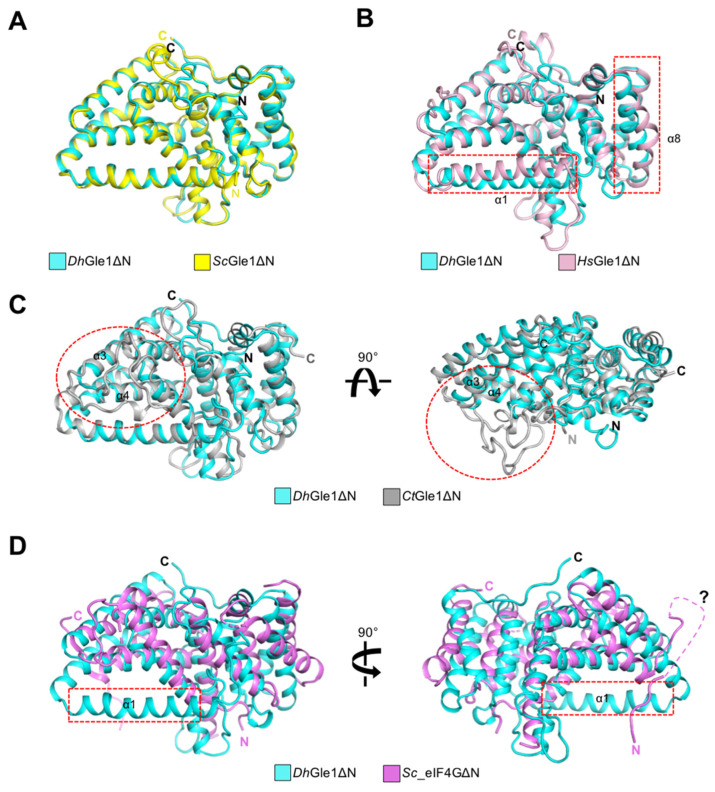
Structural overlays comparing *Dh*Gle1ΔN with four homologs: (**A**) Gle1 from *S. cerevisiae*, (**B**) Gle1 from *H. sapiens*, (**C**) Gle1 from *C. thermophilum*, and (**D**) eIF4G from *S. cerevisiae*. Regions that exhibit structural differences among the proteins are highlighted with red dashed boxes. The flexible invisible loop in the N-terminal region of *Sc*eIF4GΔN (**D**) is depicted as a magenta dashed line and is marked with a question mark.

Overall, *Dh*Gle1ΔN and the four homologous structures exhibit relatively similar folding patterns Among these, the fold of *Sc*Gle1ΔN is nearly identical to that of *Dh*Gle1ΔN, with the exception of local loops, including the N- and C-termini, resulting in an r.m.s.d. of 1.2 Å (Figure 3A, Table 1). Although *Dh*Gle1ΔN and *Hs*Gle1ΔN share similar structural characteristics, distinct conformations were observed at the α1-helix and α8-helix (Figure 3B). When comparing *Dh*Gle1ΔN to *Ct*Gle1ΔN, the loop connecting the α3-helix and α4-helix in *Ct*Gle1ΔN, which comprises 30 residues, is significantly longer than that in *Dh*Gle1ΔN (Figure 3C). On the other hand, the structure of *Sc*_eIF4GΔN diverges more markedly from that of *Dh*Gle1ΔN. Firstly, the α1-helix present in *Dh*Gle1ΔN is absent in *Sc*_eIF4GΔN; this α1-helix plays a crucial role in stabilizing the cofactor IP6. Secondly, the region spanning from the α8-helix to the α13-helix demonstrates particularly poor superimposition due to the structural differences between the two proteins. Lastly, the conformation of the N-terminal region in *Sc*_eIF4GΔN is entirely distinct from that of *Dh*Gle1ΔN (Figure 3D). A portion of the N-terminal loop (14 residues) in *Sc*_eIF4GΔN is not visible due to its inherent flexibility. Consequently, while *Sc*_eIF4GΔN does not interact with IP6, this flexible N-terminal region may potentially compensate to the IP6-independent interaction with eIF4A [6] (Figure 3D). Given its structural similarity to *Sc*_eIF4GΔN, it is plausible that *Dh*Gle1ΔN also plays a role in interactions with RNA helicase activators.

### 2.3. IP6 Binding Sites in DhGle1ΔN and Homologous Proteins

**Figure 4:**
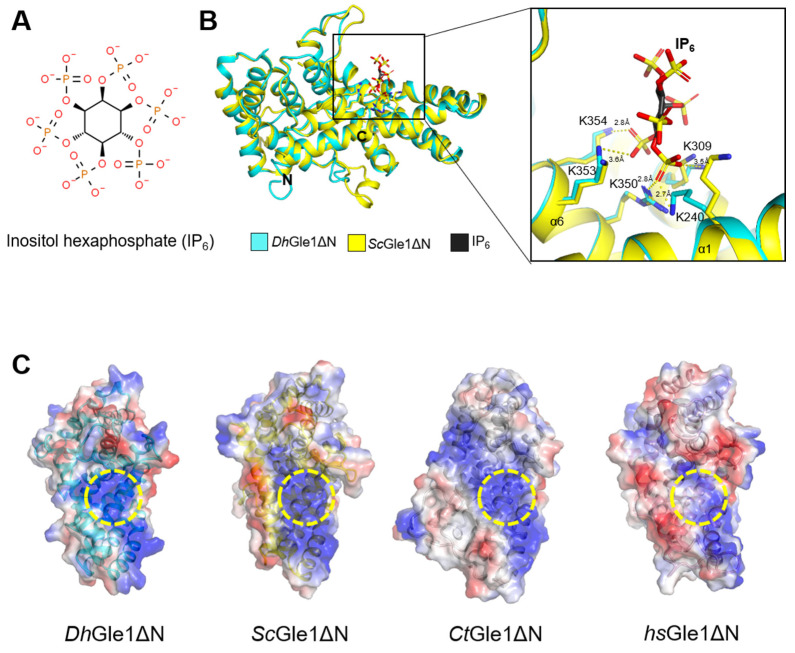
IP_6_ binding site of *Dh*Gle1ΔN. (**A**) The chemical structure of IP_6_. (**B**) Superimposed structures of *Dh*Gle1ΔN and *Sc*Gle1ΔN in complex with IP_6_ (left panel), along with a detailed view of the interactions between modeled IP6 and *Dh*Gle1ΔN (right panel). (**C**) Electrostatic surface representations of *Dh*Gle1ΔN, *Sc*Gle1ΔN, *Ct*Gle1ΔN, and *Hs*Gle1ΔN. The regions colored in red denote negatively charged surfaces, while those in blue indicate positively charged surfaces. The positively charged IP6 binding pockets of *Dh*Gle1ΔN, *Sc*Gle1ΔN, *Ct*Gle1ΔN, and *Hs*Gle1ΔN are highlighted by yellow dashed circles.

The negatively charged small molecule IP6 mediates the binding of Gle1 to Dbp5 [21] (Figure 4A). To explore the residues involved in the interaction between IP6 and *Dh*Gle1ΔN, the structure of *Dh*Gle1ΔN was superimposed with the Gle1–Dbp5 complex from *S. cerevisiae* (PDB ID: 3PEU) (Figure 4B). The basic residues Lys240, Lys309, Arg350, Lys353, and Lys354 in *Dh*Gle1ΔN interact with IP6 via hydrogen bonds or salt bridges. As expected, the IP6 binding pocket in *Dh*Gle1ΔN is highly basic, similar to those in *Sc*Gle1ΔN and *Ct*Gle1ΔN, whereas *Hs*Gle1ΔN displays a less positively charged pocket (Figure 4C). This basic pocket is conserved across fungal species but is not present in humans. Moreover, it has been demonstrated that IP6 is not required in the association of *Hs*Gle1ΔN with Dbp5 [3].

### 2.4. IP6-Dependent Complex Formation of DhGle1ΔN with DhDbp5

**Figure 5:**
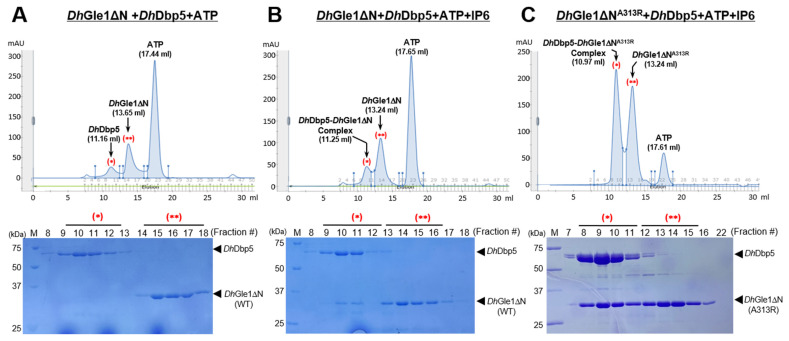
Size-exclusion chromatography (SEC) analysis of the *Dh*Gle1ΔN and *Dh*Dbp5 proteins. (**A**) SEC peak profiles (upper panel) and SDS–PAGE analysis (lower panel) of *Dh*Gle1ΔN and *Dh*Dbp5 in the presence of 10 mM ATP. The three peaks are labeled with their respective elution volumes. Fractions corresponding to DhDbp5 and DhGle1ΔN are marked with (*) and (**), respectively. The lanes are indicated as follows: M, standard molecular weight markers; 8–19, SEC-eluted fractions. (**B**) SEC peak profiles (upper panel) and SDS–PAGE analysis (lower panel) of *Dh*Gle1ΔN and *Dh*Dbp5 in the presence of 10 mM ATP and 10 mM IP6. Both *Dh*Dbp5 and *Dh*Gle1ΔN fractions are indicated by (*) and (**), respectively. The lanes are indicated as follows: M, standard molecular weight markers; 8–18, SEC-eluted fractions. (**C**) SEC peak profiles (upper panel) and SDS–PAGE analysis (lower panel) of *Dh*Gle1ΔN^A313R^ and *Dh*Dbp5 in the presence of 10 mM ATP and 100 mM IP6. Similarly, fractions for *Dh*Dbp5 and *Dh*Gle1ΔN are indicated by (*) and (**), respectively. The lanes are marked as follows: M, standard molecular weight markers; 7–16, 22, SEC-eluted fractions. All reacted proteins were subjected to SEC analysis using a Superose-12 column (GE Healthcare, Mississauga, ON, Canada) with a flow rate of 0.5 mL/min by using AK-TA Pure (GE Healthcare, Mississauga, ON, Canada). A buffer used for SEC contained 25 mM Tris-Cl (pH 7.5), 150 mM NaCl, 2 mM DTT. All the SDS-PAGE analyses were conducted using a 15% acrylamide gel with a 180 voltage for 70 min.

To evaluate the interaction between *Dh*Gle1ΔN and *Dh*Dbp5, size-exclusion chromatography (SEC) analyses were conducted. The SEC profiles showed that the main peaks for *Dh*Gle1ΔN and *Dh*Dbp5 appeared at elution volumes of 13.67 mL and 11.26 mL, respectively. To investigate complex formation, *Dh*Gle1ΔN and *Dh*Dbp5 proteins were incubated with ATP for 30 min at 4 °C, followed by SEC analysis. Based on the SEC peak profiles and SDS-PAGE results, the two proteins did not interact under these conditions (Figure 5A). The SDS-PAGE analysis clearly showed that DhGle1ΔN and *Dh*Dbp5 remained distinct, with separation observed in lanes 9 to 18. The elution volumes for *Dh*Dbp5, *Dh*Gle1ΔN, and ATP were 11.16 mL, 13.65 mL, and 17.44 mL, respectively. Subsequently, we mixed *Dh*Gle1ΔN and *Dh*Dbp5 in the presence of both ATP and IP6 for further SEC analysis (Figure 5B). The SDS-PAGE results from SEC fractions 8 to 18 indicated that the bands of DhDbp5 and *Dh*Gle1ΔN co-migrated in fractions 9 to 11 (Figure 5B, lower panel). The peak elution volumes for the *Dh*Dbp5-*Dh*Gle1ΔN complex, *Dh*Gle1ΔN, and ATP were 11.25 mL, 13.24 mL, and 17.65 mL, respectively. However, a comparison of the SEC profiles for *Dh*Dbp5 and the *Dh*Dbp5–*Dh*Gle1ΔN complex revealed an inconsistency, as *Dh*Dbp5 eluted earlier than the *Dh*Dbp5–*Dh*Gle1ΔN complex (Figure 5A,B). Additionally, the ATP peaks also exhibited differences between the two SEC profiles. This led us to hypothesize that IP6 might influence the elution volumes by altering the buffer conditions or inducing the protein’s conformational change. Furthermore, the band intensity of *Dh*Gle1ΔN in the *Dh*Dbp5 complex fraction on the SDS-PAGE indicated a weak interaction between the two proteins. Consequently, we performed SEC analysis using a gain-of-function mutation of *Dh*Gle1ΔN, specifically altering the 313rd Alanine to Arginine (*Dh*Gle1ΔN^A313R^), which corresponds to the 337th Histidine to Arginine in Gle1 from *S. cerevisiae* based on sequence alignment (Supplementary Figure S2) [6]. As anticipated, the SDS-PAGE gel results confirmed that the bands for *Dh*Dbp5 and *Dh*Gle1ΔN^A313R^ migrated together clearly (Figure 5C). Additionally, the peak positions for *Dh*Gle1ΔN^A313R^ and ATP were comparable to those observed for *Dh*Gle1ΔN and ATP in the SEC profile (Figure 5B,C). Significantly, the peak position for the *Dh*Dbp5-*Dh*Gle1ΔN^A313R^ complex shifted forward during elution, indicating that DhDbp5 and DhGle1ΔN^A313R^ form a more stable complex than *Dh*Dbp5 and *Dh*Gle1ΔN in the presence of IP6.

## 3. Discussion

The protein Gle1, in conjunction with Dbp5 and Nup159, plays a crucial role in the ATP- and IP6-dependent export of mRNA [22]. In this study, we aimed to explore the structural properties of *D. hansenii* Gle1 by revealing its crystal structure and extensively comparing it with those of homologous proteins. Initially, we used the full-length *Dh*Gle1 for structural characterization; however, this approach proved unsuccessful due to the protein’s local flexibility. Based on a previous report from the Weis group, we engineered a truncated version of the protein, excluding the N-terminal 219 residues (His6_*Dh*Gle1ΔN), which allowed for successful crystallization [6]. Despite this improvement, the crystals diffracted poorly, making it difficult to determine the structure. Following a series of optimizations, we produced a further modified version of DhGle1 lacking the 19-residue N-terminal segment containing the His6-tag (*Dh*Gle1ΔN), which diffracted exceptionally well at a resolution of 1.5 Å. This finding highlighted that the additional N-terminal segment from the artificial cloning tag was critically involved in the crystallographic packing and significantly enhanced the quality of the diffraction data obtained from the crystals.

The structures of *Sc*_eIF4GΔN and *Dh*Gle1ΔN display notable similarities, yet their functions diverge significantly [6]. Specifically, Gle1 and eIF4G interact with the RNA helicases Dbp5 and eIF4A, respectively. These binding factors serve as platforms to stimulate RNA helicase activity [23]. This raises the question: why do these cells possess two similar cofactors in the cytoplasm? To address this, it is essential to examine the role and localization of the RNA helicases. Firstly, Dbp5 functions as a shuttle between the nucleus and cytoplasm, primarily residing on the cytoplasmic side of the NPC in association with Nup159 [4]. Because Dbp5 is located at the cytoplasmic face of the NPC, it can facilitate the release of mRNA from the nucleus into the cytoplasm upon Gle1 binding [24]. In contrast, eIF4A exists in the cytoplasm, forming a complex with eIF4G that is integral to translation initiation. Secondly, although the structures of *Sc*_eIF4G and *Dh*Gle1 are largely similar, there are several variations in their local conformations. Notably, the α1-helix of *Dh*Gle1 is absent in *Sc*_eIF4G, and there are distinct differences in the regions spanning from the α8 helix to the α13 helix between the two structures (Figure 3).

In the SEC analysis, neither *Dh*Dbp5 nor *Dh*Gle1ΔN exhibited binding in the presence of ATP and MgCl_2_. However, upon the addition of IP6, the two proteins formed a complex, although SEC analysis indicated that this interaction was weak (Figure 5). What accounts for the weak IP6-dependent interaction between *Dh*Gle1ΔN and *Dh*Dbp5? First, it is worth noting that the DhDbp5 used in the SEC analysis was the full-length protein. The N-terminal region of Dbp5 may influence its interaction with *Dh*Gle1ΔN. To support the speculation, the structure of the N-terminal 111 residues of *Dh*Dbp5 was predicted using AlphaFold-2 [19]. This analysis revealed that the N-terminal region of DhDbp5 is predominantly unstructured, comprising only two short helices (Supplemental Figure S3). Consequently, this flexible and unstructured N-terminal region might destabilize the interaction with *Dh*Gle1ΔN. Further supporting this assumption, the Weis group used a truncated Dbp5 that excluded the N-terminal 90 residues, along with gain-of-function mutations in *Saccharomyces cerevisiae* Dbp5 and Gle1 (*Sc*Dbp5^L327V^ and *Sc*Gle1^H337R^), to establish the Dbp5-Gle1-ADP-IP6 complex [6,8]. Furthermore, *Dh*Gle1^A313R^, corresponding to *Sc*Gle1^H337R^, was shown to form a stable complex in the presence of IP6. Notably, Dbp5 can adopt two distinct conformations, depending on whether it is bound to ADP or ATP [6,15]. Hence, it is likely that ADP-bound Dbp5 establishes more stable interactions with *Dh*Gle1ΔN. Taken together, further investigations, including experiments using N-terminally truncated *Dh*Dbp5, ADP-bound *Dh*Dbp5, and mutations in both *Dh*Dbp5 and *Dh*Gle1, will be necessary to establish a more stable complex between *Dh*Gle1ΔN and *Dh*Dbp5 for structural studies.

Despite our advanced knowledge of mRNA export mechanisms, the intricate molecular details of these processes across diverse species remain not fully understood. To fulfill this knowledge gap, it is crucial to obtain a stable ternary complex of Gle1, Dbp5, and Nup159 from fungal species. Only by achieving this we can determine the structures of the complex, which will enable us to clarify how DhGle1ΔN recognizes and interacts with DhDbp5 and DhNup159. In future studies, given that Dbp5 also plays a role in the nuclear export of pre-ribosomal subunits and tRNAs, it will be important to investigate Gle1’s potential involvement in these processes as well [25,26]. Furthermore, exploring other aspects of nuclear export would be advantageous, as it is currently unclear which proteins are removed from the mRNP during mRNA release and how mRNA length influences the duration of the export process.

## 4. Materials and Methods

### 4.1. Cloning and Overexpression of Gle1

The gene encoding Gle1 in *D. hansenii* (NCBI ID: 2903325) was obtained from genomic DNA, and an N-terminally truncated construct was referred to as *Dh*Gle1ΔN (220–508). These genes were amplified via PCR using the forward primer 5′-AAGGCATATGACTAATTTTGCTTCTGTTGAAAA-3′ and reverse primer 5′-AAG GCTCGAGCTATGGTTCCATTTGCTTAATT-3′. The amplified fragments were digested with restriction enzymes NdeI and XhoI (Ezynomics; Daejeon, Republic of Korea), and then the digested fragments were ligated into pET28a vectors containing a 3C protease cutting site using T4 ligase (M0202S; Roche, Mannheim, Germany). The recombinant plasmids were then transformed into *E. coli* strain DH5α and confirmed by DNA sequencing. To obtain the site-directed mutant A313R, a site-specific mutation was created by PCR-based methods using Phusion high-fidelity DNA polymerase (ThermoFisher; Waltham, MA, USA) with the forward primer 5′-AAG GCT ATA ATA CGT CAA GCA G-3′ and the reverse primer 5′-CTG CTT GAC GTA TTA TAG CCT T-3′. The recombinant plasmid was sequenced to confirm correct incorporation of the mutations.

### 4.2. Purification of Recombinant Proteins

The recombinant *Dh*Gle1ΔN-pET28a-3C and *Dh*Gle1ΔN^A313R^-pET28a-3C plasmids were transformed into BL21(DE3) star cells for overexpression. Cells were grown in Luria–Bertani medium (Ambrothia, Daejeon, Republic of Korea) containing 50 mg/L kanamycin (Applichem, St. Louis, MO, USA). After reaching an optical density of 0.6, cells were induced with 0.3 mM isopropyl-β-D-thiogalactopyranoside (IPTG) at 20 °C for 18 h. The harvest cells were resuspended in a buffer containing 20 mM Tris (pH 8.0; Sigma-Aldrich, St. Louis, MO, USA), 250 mM NaCl (Applichem, St. Louis, MO, USA), 5% glycerol (Affymetrix, Santa Clara, CA, USA), 0.2% Triton X-100 (Sigma-Aldrich, St. Louis, MO, USA), 10 mM β-mercaptoethanol (BioBasic, Markham, ON, Canada), and 0.2 mM phenylmethylsulfonyl fluoride (PMSF; Sigma-Aldrich, St. Louis, MO, USA). Cells were lysed by ultrasonication (VCX-500/750; Sonics & Materials, Inc., Newtown, CT, USA) with 3 s on/off cycles continuously for 20 min. Cell debris was removed by centrifugation at 13,000 rpm for 40 min, and then the supernatant was loaded into an Ni-NTA HiTrap chelating column (GE Healthcare, Mississauga, ON, Canada) with a flow rate of 3 mL/min. After washing with a buffer (50 mM Tris, pH 8.0, 200 mM NaCl) containing 20 mM imidazole (Sigma-Aldrich, St. Louis, MO, USA), the proteins were eluted with a buffer (50 mM Tris, pH 8.0, 200 mM NaCl) containing 500 mM imidazole (Sigma-Aldrich, St. Louis, MO, USA). To remove the His_6_-tag from the expressed proteins, 250 units of 3C protease were treated at 7 °C for 12 h. The cleaved His_6_-tag was removed by additional Ni-NTA affinity chromatography. The protein was further purified using a HiPrep 16/60 Sephacryl S-300 HR column (GE Healthcare, Mississauga, ON, Canada). A buffer used for SEC contained 20 mM Tris (pH 7.5), 150 mM NaCl, and 2 mM dithiothreitol (DTT; Calbiochem, Darmstadt, Germany). The purified proteins were more than 98% pure checked by SDS-PAGE. The final concentration of the protein was 26.7 mg/mL, and it was stored at −80 °C for subsequent experiments.

### 4.3. Crystallization

Preliminary crystallizations of *Dh*Gle1ΔN were performed under more than 400 conditions using MCSG crystallization screening solution kits (Molecular Dimensions Ltd., Calibre Scientific UK, Rotherham, UK) at 7 °C. Crystals grew under 3 conditions: (1) 0.1 M CHES (pH 9.5) and 30% (*w*/*v*) PEG 3000, (2) 0.1 M BICINE (pH 9.0) and 20% (*w*/*v*) PEG 6000, and (3) Tris (pH 8.5) and 25% (*w*/*v*) PEG 3350. These three conditions were used for additional screening. Within at most five days, thick polygon-shaped crystals were obtained in drops containing equal volumes (1 µL) of the protein sample (25 mg/mL) and reservoir solution. The crystals were preserved in a cryoprotectant solution containing crystallization buffer and 30% (*v*/*v*) glycerol. The samples were flash-frozen and stored in liquid nitrogen.

### 4.4. Data Collection and Structure Determination

Diffraction datasets were collected at 100 K on a beamline 5C using a Quantum 315 CCD detector (Area Detector Systems Corporation, Poway, CA, USA) at the Pohang Accelerator Laboratory (PAL; Pohang, Republic of Korea). Crystal structures were solved by the molecular replacement method using PHENIX software version 1.9 (https://phenix-online.org/; Lawrence Berkeley Laboratory, Berkeley, CA, USA) [27]. Structural models were built using Coot [28], followed by model refinement using the PHENIX refine program. The structures were visualized using PyMOL (https://pymol.org; Schrödinger Inc., New York, NY, USA). Statistics of the data collection and refinement processes are provided in Table 2. The PDB files of Gle1 and its complex have been uploaded to the DALI web server (http://ekhidna2.biocenter.helsinki.fi/dali/, accessed on 6 November 2022) for comparison with other homologous structures using the PDB search and pairwise tools.

### 4.5. Size-Exclusion Chromatography Anaysis

The purified *Dh*Gle1ΔN, *Dh*Gle1ΔN^A313R^, and *Dh*Dbp5 full-length proteins were subjected to size exclusion chromatography (SEC) with a buffer containing 25 mM Tris-Cl (pH 7.5), 150 mM NaCl, 2 mM DTT by using AKTA Pure (GE Healthcare, Mississauga, ON, Canada), respectively. To determine whether they form a complex, incubate *Dh*Gle1ΔN (30 μM) and *Dh*Dbp5 (30 μM) proteins in the presence of either 10 mM ATP or 10 mM ATP and 10 mM IP6 for 30 min at 4 °C. The proteins *Dh*Gle1ΔN^A313R^ (50 μM), and *Dh*Dbp5 (50 μM) were also incubated in the presence of 10 mM ATP and 10 mM IP6 for 30 min at 4 °C. The reacted proteins were further applied to the Superose-12 column (GE Healthcare, Mississauga, ON, Canada) for SEC analysis. A buffer used for SEC contained 25 mM Tris-Cl (pH 7.5), 150 mM NaCl, and 2 mM DTT.

## Figures and Tables

**Table 1:** Structural homologues, identified using the DALI ^a^ server, and comparative statistics in relation to DhGle1ΔN.

Protein	Species	Z-Score	r.m.s.d. (Å)	Identity (%)	PDB Code
Gle1	*Saccharomyces cerevisiae*	41.1	1.2	49	3PEU
	*Homo sapiens*	29.8	2.3	19	6B4F
	*Chaetomium* *thermophilum*	29.5	2.2	21	6B4H
eIF4G	*Saccharomyces cerevisiae*	15.1	3.9	16	2VSX

^a^ The DALI server was employed to compute optimal and suboptimal structural alignments between two protein structures using the DaliLite-pairwise option. http://ekhidna2.biocenter.helsinki.fi/dali/, accessed on 6 November 2022.

**Table 2:** Data collection and refinement statistics for DhGle1ΔN.

Statistic	*Dh*Gle1ΔN ^a^
**Data collection**	
Space group	*P*2_1_2_1_2_1_
a, b, c (Å)	47.2, 70.7, 87.9
α, β, γ (°)	90, 90, 90
Resolution range (Å) ^a^	50–1.5 (1.55–1.50)
No. of total reflection	493,901
No. of unique reflections	47,692
Completeness (%)	99.3 (100)
*I*/σ (*I*)	49.5 (6.5)
R_merge_ (%) ᵇ	8.9 (47.8)
CC1/2	0.993 (0.927)
**Structure refinement**	
Resolution range (Å)	35.9–2.0
No. of reflections	47,608
R_work_^c^ and R_free_^d^	17.3/19.3
RMS deviation	
Bond lengths (Å)	0.007
Bond Angles (°)	0.976
Average B-factor (Å^2^)	
Protein	19.2
Solvents	30.9
Ramachandran plot ^e^	
Favored (%)	97.6
Allowed (%)	2.4
Disallowed (%)	0
PDB code	9LT9

^a^ The numbers in parentheses are statistics from the highest resolution shell. ^b^ R_merge_ = Σ|I_obs_ − I_avg_|/I_obs_, where I_obs_ is the observed intensity of the individual reflection, and I_avg_ is averaged over symmetry equivalents. ^c^ *R*−factor=∑h||*Fo*(h)|−|*Fc*(h)||/∑h|*Fo*(h)|, where Fo and Fc are the observed and calculated structure factor amplitudes, respectively. ^d^ R-free was calculated with 5% of the data excluded from the refinement. ^e^ Categories as defined by MolProbity.

## Data Availability

The original contributions presented in the study are included in the article and Supplementary Materials, further inquiries can be directed to the corresponding author.

## Supplementary Materials

The following supporting information can be downloaded at: https://www.mdpi.com/article/10.3390/ijms26041661/s1.
