# Supplementary material for: Molecular Structure of the mRNA Export Factor Gle1 from Debaryomyces hansenii
Source: Int J Mol Sci. 2025 Feb 15;26(4):1661. doi: 10.3390/ijms26041661 (PMC11855661; doi:10.3390/ijms26041661)
Supplement: Supplementary file 1 [file ijms-26-01661-s001.zip › ijms-3134102-supplementary.pdf]

Supplemental Information

Molecular Structure of the mRNA Export Factor Gle1 from *Debaryomyces hansenii*

Min Jeong Jang [1], Soo Jin Lee [1] and Jeong Ho Chang[1, 2, 3*]

1Department of Biology Education, Kyungpook National University, 80 Daehak-ro, Buk-gu, Daegu 41566, Republic of Korea

2Department of Biomedical Convergence Science and Technology, Kyungpook National University, 80 Daehak-ro, Buk-gu, Daegu 41566, Republic of Korea.

3Science Education Research Institute, Kyungpook National University, 80 Daehak-ro, Buk-gu, Daegu 41566, Republic of Korea.

*Correspondence: jhcbio@knu.ac.kr

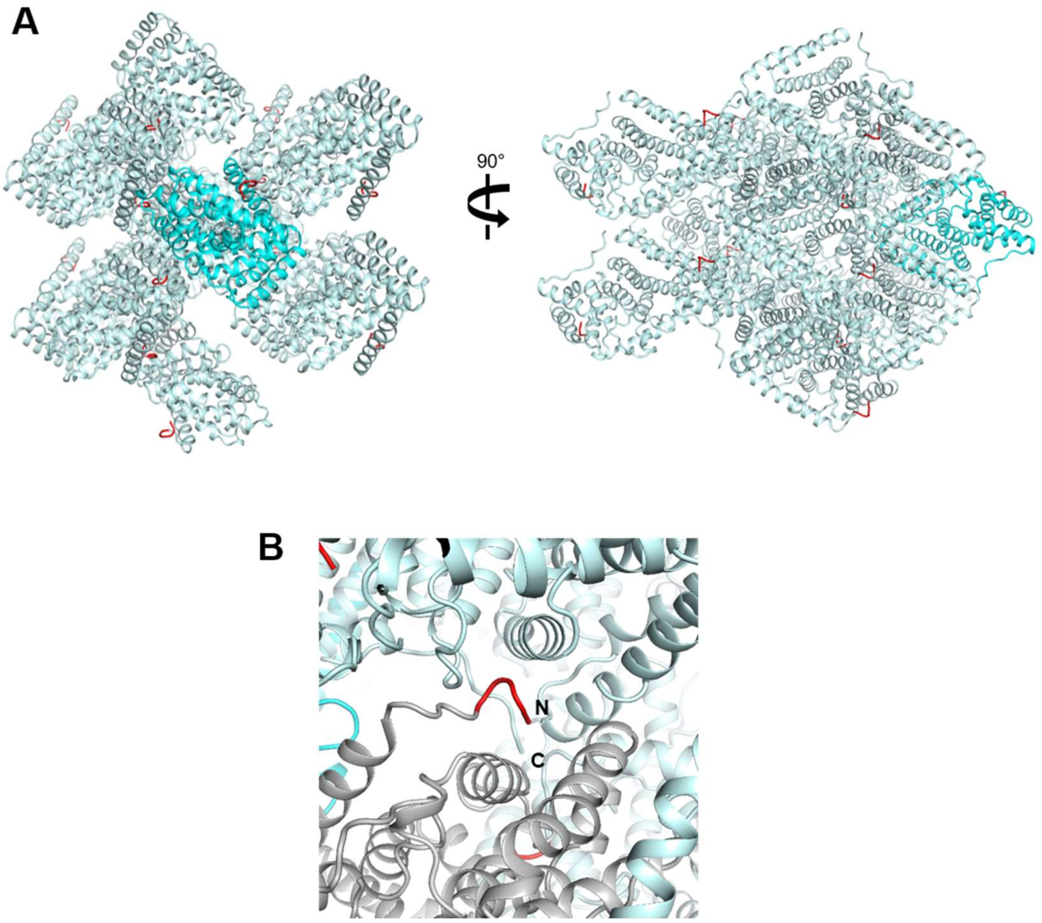

Supplemental Figure S1. (**A**) Crystallographic packing of DhGle1ΔN, with different views rotated by 90° along the y-axis. The N-terminal extra segment is colored red. (**B**) Detailed view of packing environment of N-terminal extra segment.

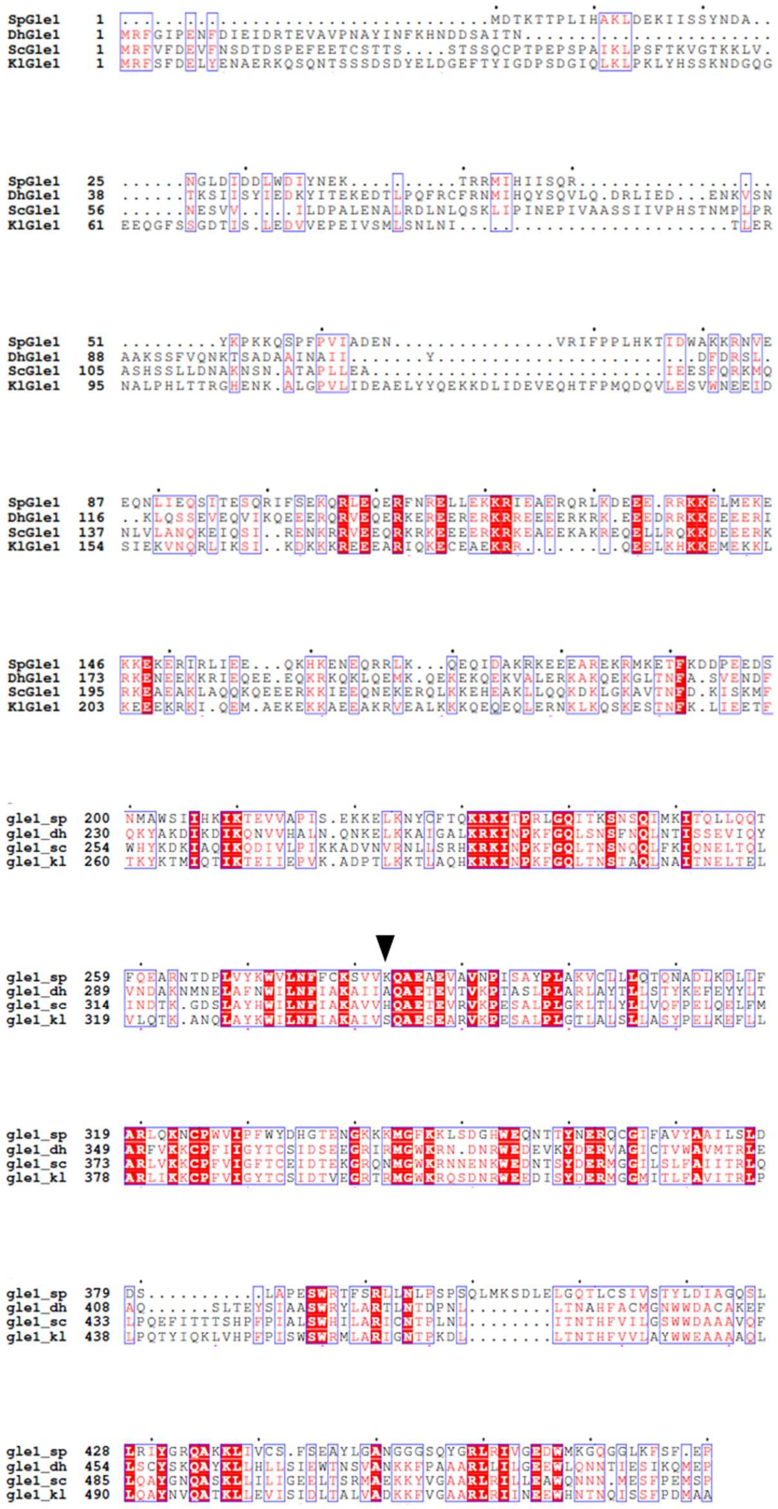

Supplementary Figure S2. The Gle1 sequences from *Schizosaccharomyces pombe* (*Sp*Gle1), *Debaryomyces hansenii* (*Dh*Gle1), *Saccharomyces cerevisiae* (*Sc*Gle1), and *Kluyveromyces lactis* (*Kl*Gle1) were compared. Conserved residues with 100% identity among the indicated species are highlighted in red, while those with 75% identity are marked with blue boxes. Black dots above the sequences indicate every 10th residue. The black arrowhead points to the location of the gain-of-function mutation point according to *Sc*Gle1.

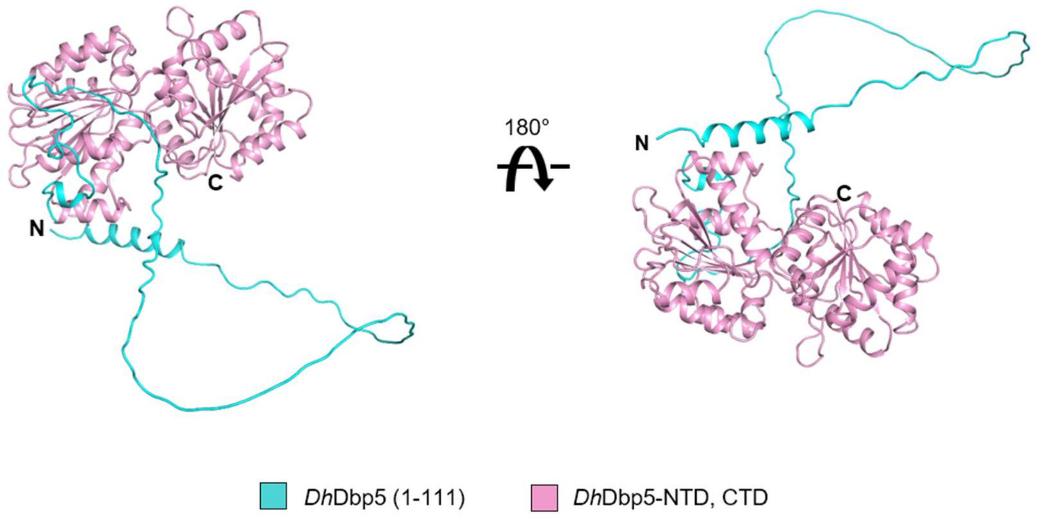

Supplemental Figure S3. The predicted structure of *Dh*Dbp5 generated by AlphaFold2. The N-terminal 111 residues show mostly unstructured loop including relatively two short a-helices.
